# Fecal HBD-2 and Claudin-3 may be potential biomarkers to predict the deterioration of necrotizing enterocolitis: A prospective study

**DOI:** 10.3389/fped.2022.1062798

**Published:** 2022-12-13

**Authors:** Xiao-Chen Liu, Lu-Quan Li, Ke-Ran Ling, Lu Guo, Xiao-Yu Hu, Chun Li

**Affiliations:** Neonatal Diagnosis and Treatment Centre of Children’s Hospital of Chongqing Medical University, National Clinical Research Center for Child Health and Disorders, Ministry of Education Key Laboratory of Child Development and Disorders, China International Science and Technology Cooperation Base of Child Development and Critical Disorders, Chongqing Key Laboratory of Pediatrics, Chongqing, China

**Keywords:** human β-defensin 2, claudin-3, biomarker, deterioration, necrotizing enterocolitis

## Abstract

**Background and purpose:**

Necrotizing enterocolitis (NEC) is a critical gastrointestinal disease. We aim to explore the value of fecal human β-defensin 2 (HBD-2), Claudin-3, high-mobility group box-1 protein (HMGB-1), and resistin-like molecule β (Relmβ) as well as some laboratory metrics to predict the deterioration of NEC.

**Methods:**

Infants diagnosed with NEC at Stage II were enrolled in our study. Those who progressed to Stage III were included in the Stage III group and the rest were included in the Stage II group. Clinical data and laboratory metrics of the infants were collected. Fecal samples of HBD2, HMGB-1, Claudin-3, and Relmβ collected during their enrollment were determined by using enzyme-linked immunosorbent assay (ELISA) kits. Student's t-test, the Mann–Whitney U test, the chi-square test, receiver operating characteristic (ROC), and logistic regression analysis were performed.

**Results:**

Sixty infants diagnosed with NEC at Stage II were enrolled in our study, with 27 in the Stage III group (*n* = 27) and 33 in the Stage II group (*n* = 33). Although many of these NEC cases were late preterm and term infants, the infants in the Stage III group had a lower gestational age (*P *< 0.05). The incidence of gestational diabetes mellitus, peritonitis, intestinal adhesion, and sepsis was higher and more infants in the Stage III group underwent surgeries (*P *< 0.05). The levels of HBD-2 and Claudin-3 were higher and neutrophil count was lower in the Stage III group than in the Stage II Group, and the area under the curve (AUC) was 0.754, 0,755, and 0.666, respectively (*P *< 0.05). HBD-2 ≥ 1649.02 ng/g and Claudin-3 ≥ 2488.71 pg/g were included in the multivariate stepwise logistic regression analysis (*P *< 0.05), and the AUC of the model was 0.805 (95% CI: 0.688–0.922).

**Conclusion:**

Fecal HBD-2 and Claudin-3 may be potential biomarkers to predict the deterioration of NEC from Stage II to Stage III.

## Introduction

Necrotizing enterocolitis (NEC) is a common and critical gastrointestinal disease in neonates, especially in preterm infants, with typical manifestations such as abdominal distention, vomiting, and blood stools (1). With the development of medical technology, the incidence of NEC has been decreasing over the past decade (2). However, it is still a serious problem in preterm infants. In those neonates born less than 32 weeks, the incidence rate ranges from 5% to 22% (3) and that of mortality ranges from 18.9% to 23.5% (4, 5). In addition, complications such as intestinal stenosis, short bowel syndrome, and neurobehavioral retardation subsequently affect the quality of life of NEC infants, especially those reaching Stage III (6, 7). Thus, for those infants with NEC, timely recognition of disease deterioration is important. However, the Bell staging criteria that are used nowadays are based on clinical manifestations and imaging presentations (8), and when infants meet the criteria of Stage III, peritonitis, septic shock, and even ensuing intestinal perforation, the disease will be life-threatening (9). Therefore, it is important to explore new indications to prevent the deterioration of NEC, especially from Stage II to Stage III.

Human β-defensin 2 (HBD-2), Claudin-3, high-mobility group box-1 protein (HMGB-1), and resistin-like molecule β (Relmβ) are potential biomarkers in intestinal inflammation, and they have been proposed as such in the diagnosis of NEC (10–15). HBD-2 is an antibacterial peptide in the innate immune system of the intestinal mucosa that can regulate the immune response with Toll-like receptor 4 and other recognition receptors to maintain the normal function that is expressed in inflammation but not in normal epithelial tissues (16–18). A previous study showed that serum HBD-2 increased in Crohn’s disease because of its systemic antimicrobial and immunomodulatory roles (19), and infants with moderate NEC showed significantly increased fecal HBD2 concentrations before the occurrence of clinical symptoms (11). Claudin-3 is an important subtype of the Claudin family, one of the most important tight conjunction proteins in the intestinal tract. It forms a dense structure between intestinal epithelial cells to reduce the permeability material exchange (20). It has been shown that Claudin-3 is a potential diagnostic biomarker for intestinal permeability and it increases in the intestinal tissues of NEC rats (10, 14).HMGB-1 in the nucleus is actively secreted by intestinal epithelial cells or inflammatory cells stimulated by inflammatory response signals or released into the intestinal lumen by necrotic cells. It can bind to non-histone chromosomes, activate Toll-like receptor 4 to inhibit intestinal cell migration, and delay the repair of intestinal mucosa (21–23). In an animal model of colitis, the expression of HMGB-1 increased in the intestinal tissue (24), and compared with those infants without NEC, the fecal level of HMGB-1 of those with NEC was significantly higher (15). Relmβ is a bactericidal protein produced by colonic epithelial goblet cells, playing an important role in the local mucosal immunity of the intestine, maintaining the integrity of the intestinal barrier, and inducing the differentiation and proliferation of epithelial cells in intestinal inflammation (25, 26). It has been pointed out that colitis mice with silencing Relmβ genes suffer less mucosa injury (27) and that serum Relmβ in neonates with NEC is significantly higher (13).

HBD2, Claudin-3, HMGB-1, and Relmβ all have potential efficacies to predict the onset of NEC (10–15). However, a few studies focused on whether they could act as potential biomarkers to predict the deterioration of NEC. In addition, the collection of fecal samples is noninvasive in nature, and compared with blood sampling, there is less pain and feces can more directly reflect intestinal injury (28). Therefore, the aim of this study is to explore the value of fecal HBD2, Claudin-3, HMGB-1, and Relmβ to predict the deterioration of NEC from Stage II to Stage III.

## Patients and methods

Infants with NEC treated in the Neonatal Diagnosis and Treatment Center at Children's Hospital of Chongqing Medical University between March 2019 and November 2020 were enrolled in this study. This study was approved by the ethics committee of Children's Hospital of Chongqing Medical University (No. 2019-284), and consent forms were obtained from parents or guardians of the infants.

### Inclusion, exclusion, and grouping criteria

Infants diagnosed with NEC at Stage II were included in our study according to the clinical manifestations or imaging presentation based on Bell's diagnostic criteria, as follows (29): (1) infants presenting with one or more clinical signs such as gastric aspirate with bile or emesis, abdominal distention, and occult and/or gross bloody stool and (2) those having at least one of the imaging findings of pneumatosis intestinalis, portal vein gas, and/or pneumoperitoneum.

The exclusion criteria were as follows: (1) infants who were diagnosed with other gastrointestinal diseases such as spontaneous intestinal perforation, Hirschsprung disease, intestinal rotation, or congenital intestinal atresia; (2) those who had cyanotic congenital heart disease, metabolic diseases, serious infections, or coagulopathy; (3) those who were discharged against medical service; (4) those who failed to complete the process of determination of fecal biomarkers; (5) those who failed to obtain consent from their guardians.

Those with disease progression to Stage III were included in the Stage III group and those who recovered from the Stage II condition were included in the Stage II group.

### Data collection and fecal sampling

Relevant information such as baseline information, disease and medication during pregnancy, and risk factors related to NEC before diagnosis were collected during their enrollment and outcomes of NEC were collected during discharge. Information on clinical manifestations and some laboratory metrics such as neutrophil count, platelet count, serum sodium, immature/total neutrophils, pH, and mean artery pressure when NEC was diagnosed was also collected.

Fecal samples were collected on the day when the enrolled infants were diagnosed with NEC at stage II. After fecal sampling, , all the enrolled infants were followed up until they were discharged for grouping. The samples were immediately sent to the laboratory and stored in a freezer at −20 °C for the detection of fecal biomarkers.

### Biomarker detection

The detection of fecal HBD2, Claudin-3, HMGB-1, and Relmβ was done by using enzyme-linked immunosorbent assay (ELISA) kits (Baijinyan Biotechnology Co., Ltd., Shanghai China) (30). Each fecal sample was allotted two wells for multiple determinations. According to the standard curve from the instructions, 50 µl of diluent and 10 µl of sample were mixed in the wells of a 96-well plate and then sealed for incubation at 37 °C for 30 min. After washing for 5 times, enzyme was added, and the incubation and wash were repeated. With the addition of chromogenic agents A and B, the plate was shaken gently for thorough mixing. Stop solution was added after keeping the plate away from drafts and other temperature fluctuations in the dark at 37 °C for 15 min. A blank well was used for zeroing, and the absorbance D (*λ*) of each well was measured in sequence at 450 nm wavelength. Taking the concentration of the standards as the abscissa and the D (*λ*) value as the ordinate, the standard curve was plotted and the regression equation was calculated. After substituting the value of the sample into the equation, the concentrations of HBD2, Claudin-3, HMGB-1, and Relmβ in feces were obtained. The lower limit of quantification was defined using the US Food and Drug Administration definition of the lowest standard with a mean accuracy of 80%–120% and a duplicate variation of <20% (31). For those samples meeting the limit, an average value of the concentration in two wells was taken or the sample discarded.

### Data analysis

All data were analyzed with SPSS statistical software (version 24; SPSS, Chicago, IL, USA). Continuous data with a normal distribution are presented as the mean ± standard deviation and were analyzed with Student's t-test. Those data with a nonnormal distribution were presented as the median (interquartile interval) and analyzed with the Mann–Whitney U test. The counting data were expressed as percentages (%) and the chi-square test was performed. The receiver operating characteristic curve (ROC) was plotted for meaningful indicators and the area under the curve (AUC) was calculated. The AUC of 0.5–0.7 was defined as low predictive value, 0.7–0.9 was defined as medium value, and a value of more than 0.9 was defined as high value (32). The best decision criteria were determined according to the best Youden index (32). To identify the value of these independent risk factors, logistic regression analysis was performed and variates were scored according to the regression coefficient in the regression analysis model (33). A score of *P *< 0.05 was considered significantly different when the data were analyzed. All charts were created with GraphPad Prism (version 9.0; GraphPad Software, Chicago, IL, USA).

## Results

During the study period, a total of 81 infants with NEC at Stage II were enrolled. Eight infants were excluded for other gastrointestinal diseases, with three for appendicitis (*n* = 3), three for gastric wall perforation (*n* = 3), and two for intestinal atresia (*n* = 2). Seven infants were discharged against medical advice (*n* = 7) and six refused to participate in this study (*n* = 6). Finally, 60 infants diagnosed with NEC at Stage II were enrolled in our study. Among them, twenty-seven progressed to Stage III rapidly and were included in the Stage III group (*n* = 27). Thirty-three infants were enrolled into the Stage II group (*n* = 33).

### Clinical features

The clinical features of infants in the Stage III and Stage II groups were compared (Table 1). The infants in the Stage III group had a lower gestational age and the incidence of gestational diabetes mellitus was higher (*P *< 0.05). Also, there was a higher rate of peritonitis, intestinal adhesion, and sepsis, more infants underwent surgeries, and hospital stays were longer in the Stage III group (*P *< 0.05). There were no significant differences between the two groups in terms of other clinical characteristics such as sex, birth weight, prenatal risk factors, and risk factors related to NEC (*P *> 0.05).

**Table 1:** Clinical features of infants in this study.

	Stage II (*n* = 33)	Stage III (*n* = 27)	*Χ^2^*/*Z*/*t*	*P*
General information				
Male, % (*n*)	58.6 (17)	41.4 (12)	0.297	0.586
Gestational age, *x* ± SD, w	35.61 ± 2.778	33.74 ± 3.404	2.345	0.022
Birth weight, *x* ± SD, g	2375 ± 638	2052 ± 707	1.861	0.068
Vaginal delivery, % (*n*)	21.2 (7)	29.6 (8)	0.561	0.454
Apgar 1 min, *M* (IQR)	9 (8,10)	10 (8,10)	−0.248	0.804
Apgar 5 min, *M* (IQR)	10 (10,10)	10 (10,10)	−0.132	0.895
Prenatal risk factors				
PROM, % (*n*)	21.2 (7)	37.0 (10)	1.831	0.176
Meconium-stained amniotic fluid, % (*n*)	6.1 (2)	7.4 (2)	0.000	1.000
Maternal hypertension, % (*n*)	12.1 (4)	11.1 (3)	0.000	1.000
Gestational diabetes mellitus, % (*n*)	3.0 (1)	33.3 (9)	7.758	0.005
ICP, % (*n*)	0.0 (0)	14.8 (4)	3.128	0.077
Antenatal steroid use, % (*n*)	27.3 (9)	44.4 (12)	1.925	0.165
Intrauterine distress, % (*n*)	9.1 (3)	11.1 (3)	0.000	1.000
Factors related to NEC before diagnosis				
Breastfeeding, % (*n*)	27.3 (9)	33.3 (9)	0.360	0.610
Antibiotic course, *M* (IQR), d	3.00 (0.00, 7.50)	2.00 (0.00, 8.00)	−0.147	0.883
Endotracheal intubation, % (*n*)	6.1 (2)	7.4 (2)	0.000	1.000
PICC usage, % (*n*)	9.1 (3)	11.1 (3)	0.000	1.000
Age of diagnosis, M (IQR), d	9.00 (5,00, 14.25)	10.00 (7.00, 22.00)	−1.495	0.135
Weight at diagnosis, *x* ± SD, g	2419.55 ± 509.945	2202.22 ± 615.188	1.497	0.140
Outcome				
Surgery, % (*n*)	0.0 (0)	66.7 (18)	31.429	0.000
peritonitis, % (*n*)	0.0 (0)	37.0 (10)	12.121	0.000
Bowel stenosis, % (*n*)	3.0 (1)	18.5 (5)	2.424	0.229
Intestinal adhesion, % (*n*)	0.0 (0)	37.0 (10)	12.121	0.000
Ileus, % (*n*)	0.0 (0)	7.4 (2)	/	0.198
Shock, % (*n*)	0.0 (0)	11.1 (3)	1.875	0.171
Sepsis, % (*n*)	42.4 (14)	81.5 (22)	9.439	0.002
Purulent meningitis, % (*n*)	0.0 (0)	14.8 (4)	3.128	0.077
Hospital stays, M (IQR), d	21.00 (14.50, 30.00)	41.00 (21.00, 56.00)	−2.930	0.003

PROM, premature rupture of membranes >18 h; ICP, Intrahepatic cholestasis of pregnancy; NEC, necrotizing enterocolitis; PICC, peripherally inserted central catheter.

### Clinical presentations

The clinical presentations of the infants during their enrollment in our study are shown in Table 2. The incidence of bloody stool, vomiting, abdominal distention, abdominal wall erythema, absent bowel sounds, and abdominal wall edema showed no significant difference (*P *> 0.05).

**Table 2:** Clinical presentations of the infants in the two groups during their enrollment.

	Stage II (*n* = 33)	Stage III (*n* = 27)	*Χ^2^*	*P*
Bloody stool, % (*n*)	72.7 (24)	77.8 (21)	0.202	0.653
Vomiting, % (*n*)	15.2 (5)	7.4 (2)	0.276	0.599
Abdominal distention, % (*n*)	90.9 (30)	88.9 (24)	0.000	1.000
Abdominal wall erythema, % (*n*)	0.0 (0)	11.1 (4)	1.875	0.171
Absent bowel sounds, % (*n*)	84.8 (28)	88.9 (24)	0.006	0.939
abdominal wall edema, % (*n*)	0.0 (0)	14.8 (4)	3.128	0.077

### Laboratory metrics

**Figure 1:**
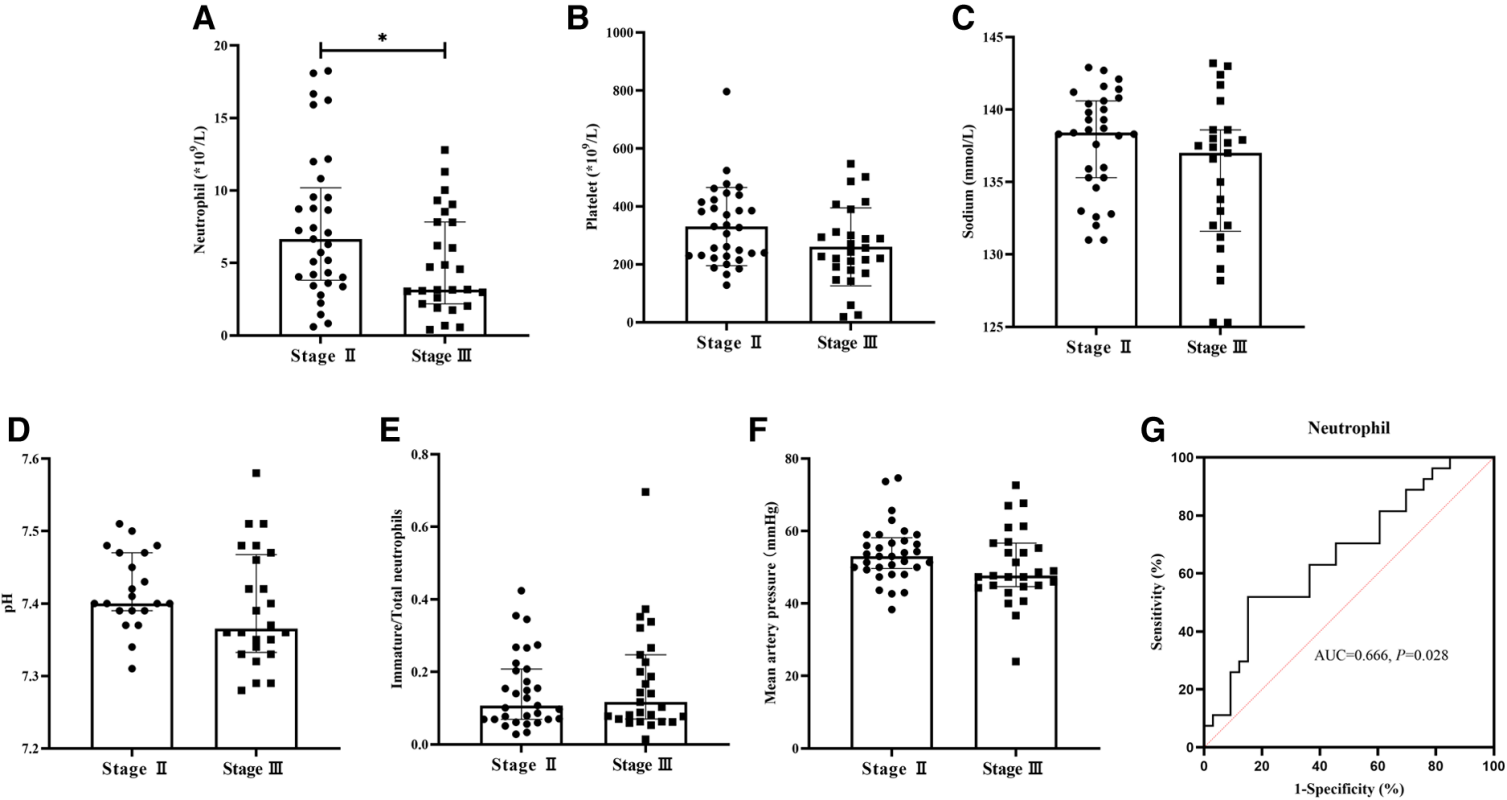
Comparison of laboratory metrics between two groups and ROC of neutrophil count. Neutrophil count in the Stage III group significantly decreased (*P *< 0.05). (**A**) Platelet count, serum sodium, pH, immature/total neutrophils, and mean artery pressure showed no differences between the two groups (*P *> 0.05). (**B–F**) The AUC of neutrophil count was 0.666 (*P *< 0.05) (**G**).

Laboratory metrics were compared in Figure 1. In the Stage III group, neutrophil count [(3.17 (2.18, 7.83) vs. 6.65 (3.81, 10.19), × 10^9^/L, *P *= 0.028] when the infants were diagnosed with NEC was significantly lower (Figure 1A). Platelet count [(250.42 ± 129.694) vs. (330.33 ± 135.026), × 10^9^/L, *P *= 0.050], serum sodium [(137.00 (131.60, 138.60) vs. 138.40 (135.30, 140.60), mmol/L, *P *= 0.079], immature/total neutrophils [0.12 (0.07, 0.25) vs. 0.11 (0.07, 0.21), *P *= 0.680], pH [(7.39 ± 0.080) vs. (7.42 ± 0.053), *P *= 0.242], and mean artery pressure [(50.17 ± 10.313) vs. (53.91 ± 7.890), mmHg, *P *= 0.117] showed no difference (Figures 1B–F). The AUC of neutrophil count was 0.666 (Figure 1G).

### Fecal biomarkers

**Figure 2:**
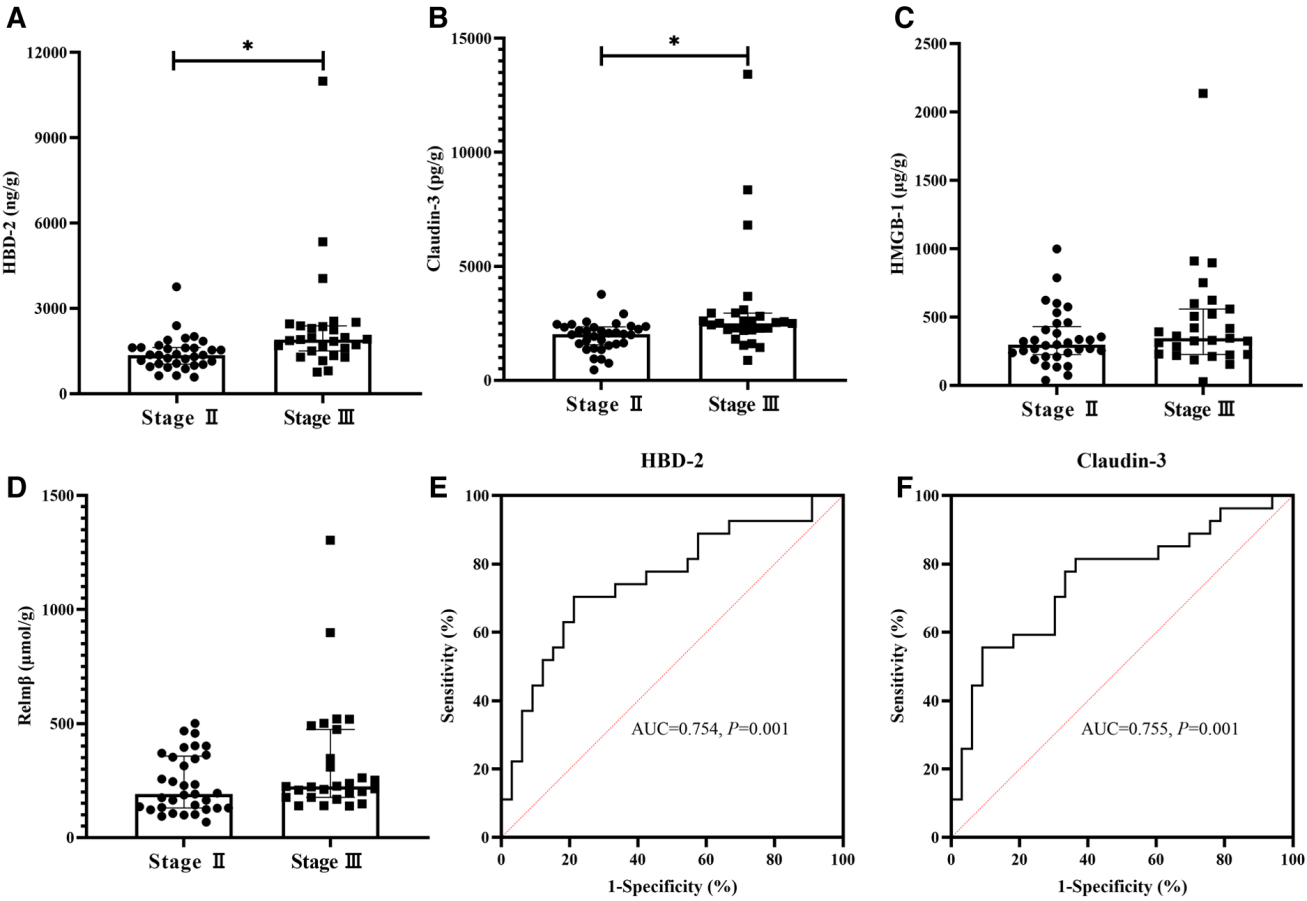
Comparison of fecal biomarkers between two groups and ROCs of HBD-2 and Claudin-3. The fecal concentrations of human β-defensin 2 (HBD-2) and Claudin-3 were significantly higher in the Stage III group than in the Stage II Group (*P *< 0.05) (**A,B**). High-mobility group box-1 protein (HMGB-1) and resistin-like molecule β (Relmβ) showed no differences between the two groups (*P *> 0.05) (**C,D**). The AUC of HBD-2 and Claudin-3 were 0.754 and 0,755, respectively (*P *< 0.05) (**E,F**).

A comparison of fecal biomarkers and their ROC curves are shown in Figure 2. As for the fecal biomarkers, HBD-2 [1901.71 (1500.65, 2383.81) vs. 1358.09 (1038.25, 1621.99), ng/g, *P *= 0.001], and Claudin-3 [2504.09 (2211.13, 2942.50) vs. 2023.50 (1557.50, 2342.09), pg/g, *P *= 0.001] in the Stage III group were significantly higher than those in the Stage II group (Figures 2A,B). The fecal biomarkers of HMGB-1 [346.00 (226.44, 558.42) vs. 297.50 (224.75, 430.10), µg/g, *P *= 0.226] and Relmβ [224.06 (176.50, 474.00) vs. 190.74 (129.55, 357.25), µmol/g, *P *= 0.064] showed no differences between the two groups (Figures 2C,D). The AUC of HBD-2 and Claudin-3 were 0.754 and 0,755, respectively (Figures 2E,F). According to the best Youden index, the cutoff of HBD-2 was defined as 1649.02 ng/g with a sensitivity of 70.4% and specificity of 78.8% and the cutoff of Claudin-3 was 2488.71 pg/g with a sensitivity of 55.6% and specificity of 90.9%.

### Predictive scores

**Figure 3:**
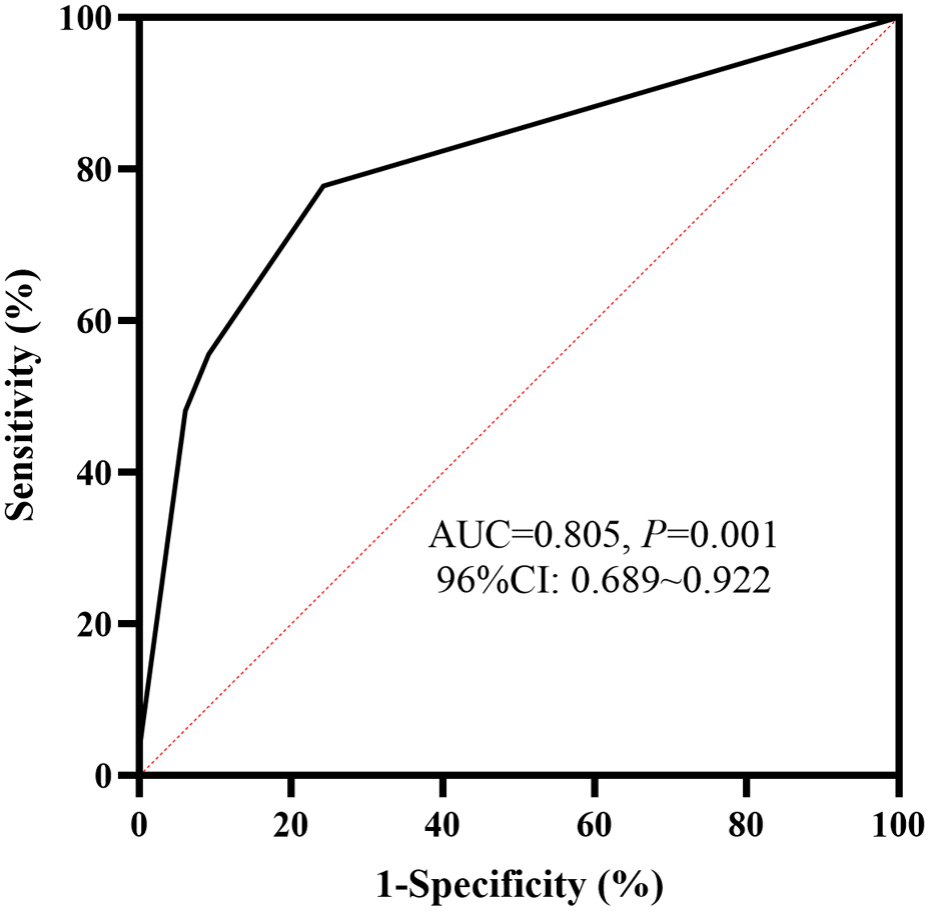
ROC curve of fecal biomarkers. The AUC of human β-defensin 2 (HBD-2) ≥ 1649.02 ng/g, combined with Claudin-3 ≥ 2488.71 pg/g, to predict the deterioration of NEC was 0.805, and it showed a statistical significance (*P *< 0.05). ROC, receiver operating characteristic; AUC, area under the curve; NEC, necrotizing enterocolitis.

HBD-2 ≥ 1649.02 ng/g, Claudin-3 ≥ 2488.71 pg/g, and neutropenia (defined as ≤ 1.5 × 10^9^/L) (34) were included in the multivariate stepwise logistic regression analysis. HBD-2 ≥ 1649.02 ng/g and Claudin-3 ≥ 2488.71 pg/g were independent predictive factors of the deterioration of NEC from Stage II to Stage III and the model showed a significant difference (*P *< 0.05). According to the regression coefficient in Table 3, HBD-2 ≥ 1649.02 ng/g and Claudin-3 ≥ 2488.71 pg/g scored 1.516 and 1.848 points, respectively, to obtain the predictive scores. The obtained scores in the Stage III group were higher than those in the Stage II group [1.848 (1.516, 3.364) vs. 0.000 (0.000, 0.758), *P *= 0.000], and the AUC in the ROC curve of the obtained scores was 0.805 (95% CI: 0.688–0.922), which showed a medium value to predict the deterioration of NEC from Stage II to Stage III (Figure 3).

**Table 3:** Multivariate analysis of biomarkers to predict the deterioration of NEC.

Variables	*β*	SE	Wald	*P*	OR	95%CI
HBD-2 ≥ 1649.02 ng/g	1.516	0.666	5.182	0.023	4.552	1.235–16.783
Claudin-3 ≥ 2488.71 pg/g	1.848	0.782	5.592	0.018	6.349	1.372–29.373

## Discussion

NEC is a severe intestinal disease in neonates (3) and it is important to recognize the progression of diseases in time to reduce mortality and the incidence of sequelae, including peritonitis, intestinal adhesion, and sepsis, as shown in our study (35). We investigated the value of fecal biomarker as well as some non-specific laboratory metrics in the progression of NEC from Stage II to Stage III and found that fecal HBD-2 and Claudin-3 may be potential biomarkers in the prediction of deterioration of NEC.

Jenke et al. found that HBD-2 increased in the intestinal tissues from very-low-birth weight infants in moderate NEC, while in six infants with severe NEC, HBD-2 decreased when surgeries were performed on them (11). Thuijls et al. explored the urinary Claudin-3 in 14 NEC infants and found that it could be promising biomarkers for diagnosis, but they did not possess the data of urine Claudin-3 in severe NEC infants (36). To our knowledge, few clinical studies have focused on fecal HBD-2 and Claudin-3 to predict the disease progression of NEC. In our study, we found that when the infants were diagnosed with NEC, the levels of HBD-2 and Claudin-3 were higher in those who finally progressed to Stage III. Moreover, in our study, ROC curves were established and both showed medium value to predict the deterioration of NEC, and we also evaluated the combined utility of HBD-2 and Claudin-3. We found that when the fecal concentrations of both HBD-2 and Claudin-3 increased once NEC was diagnosed at Stage II, there was a high possibility of Stage II progressing to Stage III. Thus, to prevent disease progression, when HBD-2 concentration is higher than 1649.02 ng/g and Claudin-3 concentration is over 2488.71 pg/g, in the infants, extra attention should be paid to them and positive treatment should be given clinically.

Also, in our study, neutrophil count was found decreased in infants progressing to Stage III. However, in the advanced regression analysis, when we adapted the widely accepted standard of neutropenia as an independent risk factor, it showed no significant difference. Thus, the value of neutrophil as well as its cutoff value in the prediction of deterioration of NEC should be further explored in clinical trials with larger sample sizes in the future. In addition, we measured the fecal concentration of HMGB-1 and Relmβ in our study. Fecal HMGB-1 has been reported to be helpful in the diagnosis of NEC (15), and our previous study found that in those needing surgeries, the level of serum Relmβ was even higher (12). HMGB-1 participates in intestinal inflammation of NEC by inhibiting the NLR pyrin domain containing 3 via Toll-like receptor 4 and nuclear factor-kappaB signaling pathways and decreases intestinal microcirculatory perfusion by rescuing nitric oxide production and eliminating oxygen production through endothelial nitric oxide synthase activation (23, 37). Thus, HMGB-1 might increase in NEC, in which both infection and hypoxia play an important role. Relmβ is a bactericidal protein that is increasingly secreted in intestinal inflammation expression in the tissue (38). Relmβ stimulates resident immune cells within the mucosa to secrete significant amounts of proinflammatory cytokines by nuclear factor kappaB in ileitis (39, 40) and takes part in the maintenance of the mucosal defense barrier in colitis by upregulating the gene expression of mucin between intestinal epithelial cells (41). However, HMGB1 and Relmβ showed no statistically significant difference. The small sample size might be the main reason for this, warranting further exploration in clinical studies with larger sample sizes.

There were some limitations in our study. First, fecal biomarkers should be dynamically monitored in the progression from Stage II to Stage III. Second, there are several outliers of the biomarkers and they might impact the present results of our study with their small sample sizes. Therefore, a larger sample size study is needed to determine whether these outliers represent the internal changes of these biomarkers. Third, the gestational age of the infants in the Stage III group was smaller than that of those in the Stage II group. It has been found that concentrations of fecal HBD-2 increase with increasing gestational age (42), and it seems that it was not influenced by the difference of gestational age in our study. However, it has not been reported how fecal claudin-3 changes with gestational age in neonates, and whether gestational age is a significant confounder in our study still needs further exploration. Finally, as the center of the Neonatal Emergency Transport System in southwestern China, our department serves neonates form several provinces, and many of the NEC patients enrolled in our study were late preterm and term infants, because most infants admitted to our department were of larger gestational age. Thus, multicentral clinical trials with larger sample sizes enrolling infants with different gestational ages, especially preterm or extremely preterm infants, are needed to verify the predictive value of these biomarkers in the study.

## Conclusion

In this study, we found that fecal HBD-2 and Claudin-3 increased, while neutrophil decreased when NEC infants progressed to a worsening condition. Fecal HBD-2 and Claudin-3 may be potential biomarkers to predict the deterioration of NEC from Stage II to Stage III, and when their levels increase in some patients, more attention should be paid to them.

## Data Availability

The raw data supporting the conclusions of this article will be made available by the authors, without undue reservation.
